# Agronomic Traits, Nutrient Accumulation, and Their Correlations in Wheat, as Affected by Nitrogen Supply in Rainfed Coastal Saline Soils

**DOI:** 10.3390/plants14071022

**Published:** 2025-03-25

**Authors:** Yan Li, Shuaipeng Zhao, Guolan Liu, Jian Li, Kadambot H. M. Siddique, Deyong Zhao

**Affiliations:** 1 College of Biological and Pharmaceutical Engineering, Shandong University of Aeronautics, Binzhou 256603, China; 23021201005@sdua.edu.cn (Y.L.);; 2 Shandong Key Laboratory of Eco-Environmental Science for Yellow River Delta, Shandong University of Aeronautics, Binzhou 256603, China; 3 Shandong Engineering and Technology Research Center for Fragile Ecological Belt of Yellow River Delta, Shandong University of Aeronautics, Binzhou 256603, China; 4 The UWA Institute of Agriculture, The University of Western Australia, Perth, WA 6001, Australia; kadambot.siddique@uwa.edu.au

**Keywords:** nitrogen fertilization, coastal areas, soil salinity, selenium, *Triticum aestivum*

## Abstract

How nitrogen (N) levels affect agronomic performance and the nutrient utilization process in wheat grown in rainfed coastal saline soils remains largely unknown. This study investigated the influence of three N supply treatments (0, 100, and 200 kg/ha) on the growth and accumulation of P, Ca, Mg, K, Na, Zn, Fe, and Se of eight wheat genotypes across two consecutive seasons (2020–2021, 2021–2022) in a rainfed coastal field. Both agronomic performance and nutrient accumulation were significantly affected by N supply and genotypic effects. The increased total accumulation of nutrients was mainly due to enhanced agronomic performance by N supply. Grain Zn and Fe concentrations increased, while the grain Se concentration decreased with the N supply increasing. Genotype “Jimai 775” exhibited both a higher grain yield and a higher nitrogen agronomic efficiency among the tested genotypes. The association among agronomic traits and nutrient accumulation was obviously modified by the N supply, as revealed by principal component analysis, correlation analysis, and stepwise multiple regression models. These findings suggest that both the N supply level and genotypic differences should be taken into consideration to enhance nutrient utilization in wheat cultivated in coastal saline soils.

## 1. Introduction

Globally, salinity affects about 17 million km^2^ of land [1]. Coastal soils, particularly those in semi-arid and arid climates, often exhibit elevated salt content (measured as electrical conductivity, EC) and/or higher pH due to shallow groundwater tables, limited rainfall, and high evaporation rates [2]. Consequently, these coastal saline soils frequently suffer from compaction, hindering the efficient transfer of nutrients from soil to crops. Successful crop production in such environments necessitates the development of salt-tolerant crop varieties and improved fertilizer management practices.

Throughout their entire growth periods, wheat plants require a range of macronutrients and micronutrients, accumulating these nutrients in their grains, which are vital food sources for humans. Essential micronutrients support various physiological and biochemical processes within organisms, although they account for only a small portion of the dry matter. Inadequate Zn supply intake can lead to growth delays, cell-mediated immune dysfunction, and cognitive impairment [3], while Fe deficiency results in anemia. The National Institute of Health (USA) recommends 8–11 mg daily Zn for adults, while the average daily dietary Fe intake typically ranges from 10 to 15 mg [4]. The inadequate intake of Se in humans is also prevalent; the recommended dietary intake of selenium (Se) is 50–55 µg day [5]. Crop grains with higher available beneficial micronutrients such as Zn, Fe, and Se are required to sustain a sufficient intake by the human body. However, the Zn and Fe concentrations in wheat grain worldwide often fall short of biofortification targets, set at 40 mg/kg for Zn and 60 mg/kg for Fe [6,7,8]. The Se concentration in wheat grain varied largely across investigated sites, with an average of 0.03–0.16 mg/kg [9]. Significant progress has been made in breeding Zn- and Fe-enriched wheat varieties, with genetically biofortified wheat lines exhibiting 75–150% higher grain Zn content than non-biofortified counterparts [10]. Additionally, agronomic approaches, such as soil or foliar application of fertilizers at appropriate growth stages, are commonly used to enhance Zn and Se accumulation [11,12].

The N supply level is a critical factor influencing wheat grain yield and protein concentration. Proper soil N levels can facilitate the uptake of other essential elements, including phosphorus (P), calcium (Ca), and boron (B) [13]. Moreover, a positive correlation exists between the grain protein concentration and Zn and Fe concentrations, as grain protein is a sink for these micronutrients. Thus, N supply can increase grain protein, Zn, and Fe accumulation [14,15]. However, the extent to which N supply enhances the whole grain Zn concentration depends on the initial soil Zn content, with a high N supply increasing the grain Zn concentration only when soil Zn levels are high [16,17]. A nitrogen fertilizer rate of 120–240 kg/ha had increased concentrations of water-soluble Se and exchangeable Se in soil, but a higher N rate exceeding 240 kg/ha could hinder the formation of soil-available Se [18]. On the other hand, the application of Se could also improve N metabolism in wheat [19].

Interactions between elements can exhibit antagonistic or synergistic effects, influencing plant ion uptake and translocation. For instance, Zn positively correlated with N, potassium (K), magnesium (Mg), and manganese (Mn) in wheat seedlings [20]. Conversely, high P supply and soil Fe levels can limit Zn accumulation in wheat grain [21,22]. Plant Fe deficiency may occur with liming (application of Ca- and Mg-rich materials), as Fe and Mg exhibit mutual antagonism [23]. Furthermore, inadequate K supply or excessive Zn levels can also lead to Fe deficiency [13]. Soil properties and agronomic practices including N supply, together with antagonistic and/or synergistic effects among elements, affect the final concentration of a specific element within wheat plants at maturity. Understanding how crop growth would be affected by soil properties and agronomic measures is of importance to explore coastal soils for agricultural purposes. To date, limited information is available on the effects of N supply on K, Ca, Mg, P, Zn, Fe, and Se accumulation in wheat grown under coastal soil conditions. This study was, therefore, conducted to (1) explore the impact of N supply on agronomic performance and nutrient accumulation; (2) elucidate the associations between agronomic performance and nutrient accumulation, as mediated by N supply under rainfed coastal soil conditions.

## 2. Results

### 2.1. Agronomic Performance of Wheat Genotypes in Response to N Supply

Agronomic traits, including plant height, fertile spikelet number per head, grain number per head, 1000-kernel weight, grain yield, and straw weight, were significantly affected by genotype, N supply, and their interaction (Supplementary Table S1). The investigated agronomic traits, excluding straw weight, varied significantly between growth seasons. Generally, the N200 treatment resulted in the highest values for plant height, fertile spikelet number, grain number per ear, grain yield, and straw weight in both seasons (Table 1). Agronomic performance also varied between seasons, with the mean grain number increasing from 42.5 in 2020–2021 to 44.2 in 2021–2022, fertile spikelet number per head increasing from 15.6 in 2020–2021 to 16.2 in 2021–2022, 1000-kernel weight increasing from 40.67 g in 2020–2021 to 41.50 g in 2021–2022, and grain yield increased by 4.49% in 2021–2022 compared to 2020–2021.

Significant differences occurred among the eight genotypes for plant height, fertile spikelet number per head, grain number per head, 1000-kernel weight, grain yield, and straw weight (Table 2). Across all three N supply treatments, Jimai 60 recorded the tallest plants (average 63.9 cm), while Hongdi 95 had the shortest (average 50.6 cm). Fertile spikelet number per head ranged from 15.2 (LS018R) to 16.9 (Jimai775), grain number per head ranged from 40.2 (LJJ803) to 45.3 (Jimai60), and 1000-kernel weight ranged from 37.2 (Jimai775) to 44.1 (Jimai60). Genotypes Jimai60 and Jimai775 consistently achieved significantly higher average grain yields than the other six genotypes across all three N treatments. In the N0 treatment, Jimai60 and Jimai775 yielded 4899.8–5099.1 kg/ha and 4133.7–4334.0 kg/ha, respectively.

### 2.2. Nutrient Accumulation

**Figure 1:**
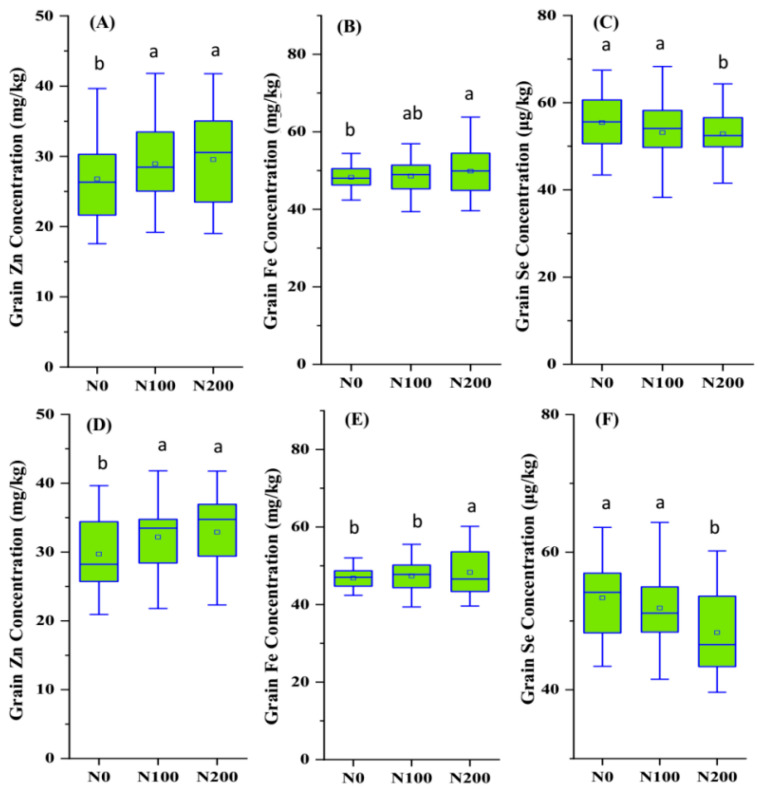
Grain (**A**,**D**) Zn, (**B**,**E**) Fe, and (**C**,**F**) Se concentrations in eight wheat genotypes under three N supply levels (N0, N100, N200) in the (**A**–**C**) 2020–2021 and (**D**–**F**) 2021–2022 seasons. Note: Different letters indicate statistically significant difference (*p* < 0.05).

ANOVA results revealed significant effects of season, genotype, and N supply on Na, Mg, P, K, Ca, Zn, Fe, and Se concentrations in both grain and straw. Grain had significantly lower Na, K, Ca, and Fe concentrations and higher P and Zn concentrations than straw. On average, grain had a 305.0% higher Zn concentration and 74.5% lower Fe concentration than straw. Grain Zn concentrations showed an increased trend with the N supply increasing (Figure 1A,D); the average values under N0, N100, and N200 treatments were 26.8 mg/kg, 28.9 mg/kg, and 29.5 mg/kg, respectively. Similarly, the N supply significantly increased the grain Fe concentration, with the highest average achieved under N200 treatment (Figure 1B,D). Inversely, the grain Se concentration showed a decreased trend with the N supply increasing (Figure 1C,F); the average values under N0, N100, and N200 treatments were 55.4 μg/kg, 54.7 μg/kg, and 53.1 μg/kg, respectively.

Moreover, significant genotypic differences were observed in grain elemental concentrations (Table 3). For instance, the grain Zn concentration ranged from 23.7 mg/kg (LS3666) to 33.1 mg/kg (Jimai 106), grain Fe concentration ranged from 46.8 mg/kg (Jimai 775) to 61.9 mg/kg (LJJ803), and grain Se concentration ranged from 48.8 μg/kg (Jimai106) to 58.6 μg/kg (Shannong25) among the eight genotypes grown under three N supply levels. The total elemental accumulation of P, K, Ca, Mg, Zn, Fe, and Se in grain and straw increased in the N100 and N200 treatments relative to N0, mainly due to increased grain yield and straw weight.

Aboveground biomass accumulation partly relies on nutrient uptake from the soil. Enhanced agronomic traits observed in the N100 and N200 treatments resulted in higher grain yield and straw weight than the N0 treatment, accompanied by increased grain and straw P, K, Ca, and Mg accumulation. Moreover, significant genotypic differences were observed in response to N supply levels, with Jimai 775 and Jimai 60 producing higher grain yield under low-N supply.

### 2.3. Nitrogen Agronomic Efficiency of Eight Wheat Genotypes

NAE, an index evaluating the response of grain yield to the N supply amount, was used to examine the difference among genotypes, N treatments, and seasons (Table 4). NAE was affected significantly by N treatment, genotype, and season. In the 2020–2021 season, NAE varied from 0.5 (LS3666) to 18.7 (Jimai775) in the N100 treatment and from 3.3 (LS018R) to 15.4 (Jimai775) in the N200 treatment. In the 2021–2022 season, NAE varied from 3.8 (LS018R) to 16.0 (Jimai775) in the N100 treatment and from 2.2 (LS3666) to 20.4 (Jimai775) in the N200 treatment. ‘Jimai 775’ and ‘Shannong 25’ achieved the highest NAE among the eight tested wheat genotypes in both the N100 and N200 treatments across both seasons.

### 2.4. Associations Between Agronomic Performance and Nutrient Accumulation

**Figure 2:**
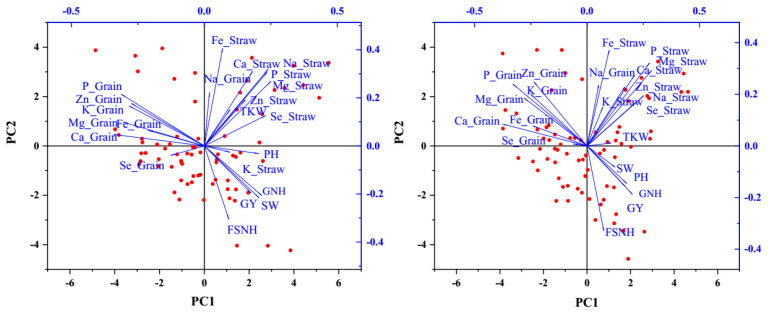
PCA plots for the (**left**) 2020–2021 and (**right**) 2021–2022 wheat growing seasons. Abbreviations: PH, plant height; GY, grain yield; SW, straw weight; GNH, grain number per head; FSNH, fertile spikelet number per head; TKW, 1000-kernel weight; P_grain, grain P concentration; Mg_grain, grain Mg concentration; K_grain, grain K concentration; Na_grain, grain Na concentration; Ca_grain, grain Ca concentration; Zn_grain, grain Zn concentration; Fe_grain, grain Fe concentration; P_straw, straw P concentration; Mg_straw, straw Mg concentration; K_straw, straw K concentration; Na_straw, straw Na concentration; Ca_straw, straw Ca concentration; Zn_straw, straw Zn concentration; Fe_straw, straw Fe concentration; Se_straw, straw Se concentration.

**Figure 3:**
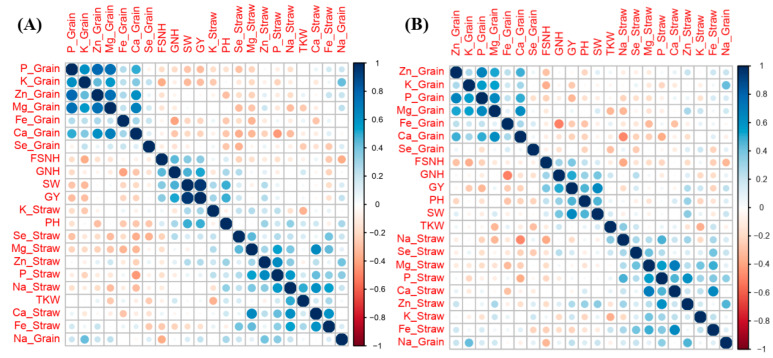
Correlations between agronomic trait and nutrient concentration in the 2020–2021 (**A**) and 2021–2022 (**B**) wheat growing seasons.

The PCA explored associations between agronomic traits and nutrient accumulation. For the eight genotypes, the PCA explained 38.42% of the variance in the first two components in the 2020–2021 season and 34.10% in the 2021–2022 season (Figure 2). The first component (PC1) accounted for 22.40% of the variability in the 2020–2021 season and 18.16% in the 2021–2022 season, primarily representing agronomic traits. The second component (PC2) explained 16.02% of the variability in the 2020–2021 season and 15.94% in the 2021–2022 season, mainly accounting for elemental grain and straw concentrations. Three distinct clusters representing agronomic traits, elemental accumulation in straw, and elemental accumulation in grain appeared separately in the PCA plots, with components within the same cluster positively correlated. To be more specific, correlation maps involving agronomic traits and nutrient concentration were drawn for both growing seasons (Figure 3). The grain yield positively correlated with plant height, straw weight, fertile spikelet number per head, and grain number per head in both seasons. The grain Zn concentration positively correlated with the grain Mg concentration, grain P concentration, grain K concentration, grain Ca concentration, and grain Fe concentration in both seasons. The straw Zn concentration and straw Fe concentration positively correlated with the straw Na concentration, straw Mg concentration, straw P concentration, and straw Ca concentration. The grain Se concentration negatively correlated with plant height, 1000-kernel weight, straw Se concentration, and straw Fe concentration.

**Figure 4:**
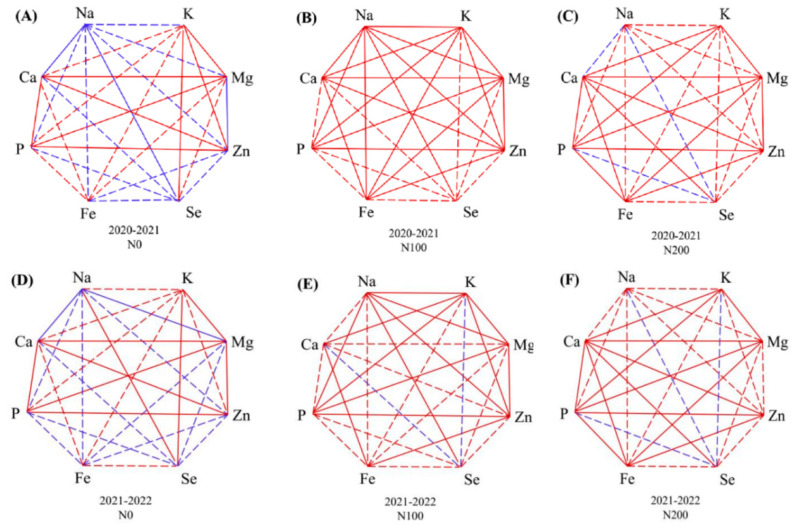
Correlations among grain elemental accumulation under (**A**,**D**) N0, (**B**,**E**) N100, and (**C**,**F**) N200 treatments during (**A**–**C**) 2020–2021 and (**D**–**F**) 2021–2022 seasons. Note: Blue and red lines indicate negative and positive correlations, respectively. Bold and dash lines indicate correlations are significant and non-significant, respectively.

Further correlations among grain elemental accumulation under N0, N100, and N200 treatments were examined (Figure 4). Overall, the correlation among grain elemental accumulation was significantly changed by the N supply. In addition, the correlation also varied between seasons. For example, the correlation between the grain Zn concentration and grain Na concentration was non-significantly negative, significantly positive, and non-significantly positive under the N0, N100, and N200 treatments, respectively. The grain Zn concentration was consistently positively correlated with the grain P concentration across all N treatments in both seasons. Grain Fe significantly positively correlated with grain P and Zn concentration under the N100 and N200 treatments.

Stepwise multiple regression models were further applied to construct equations using the investigated variables. The grain Zn concentration, grain Fe concentration, and grain Se concentration can be explained by some of the investigated agronomic traits and some elemental concentrations in straw and grain (Supplementary Table S2). Equations varied slightly among different N treatments and across seasons. Generalized multiple regression equation models were established using combined data from all treatments in both the 2020–2021 and 2021–2022 seasons. The generalized multiple regression equation model revealed that the grain Zn concentration can be predicted using a regression equation that includes the grain P concentration, grain Ca concentration, straw weight, plant height, straw Zn concentration, 1000-kernel weight, and grain Mg concentration. Similarly, the grain Fe concentration can be predicted using a regression equation that includes straw P concentration, grain K concentration, fertile spikelet number per head, grain number per head, straw Mg concentration, and plant height. The grain Se concentration can be predicted using a regression equation that includes straw Se concentration, plant height, grain Na concentration, grain P concentration, grain Mg concentration, grain K concentration, grain Ca concentration, and straw Na concentration.

## 3. Discussion

### 3.1. Seasonal Variation of Agronomic Performance and Nutrient Utilization Under Coastal Soil Conditions

Crop growth in a rainfed coastal field of the Yellow River Delta region, China, could be hampered by both soil properties and saline ground water, as roots penetrate the deeper soil layer. On the other hand, weather conditions, particularly rainfall distribution in arid and semi-arid rainfed fields, play a crucial role in soil moisture availability, influencing crop reactions in root system development due to nutrient availability [24]. In dry seasons, limited rainfall results in frequent topsoil drying, restricting nutrient uptake. Moreover, the movement of topdressed N into deeper soil layers is hindered, further limiting N uptake by deeper roots. Conversely, wet seasons promote greater root system development [25], facilitating N uptake from topsoil and deep soil. Consequently, N supply effectiveness can vary based on seasonal conditions, with wet years typically yielding better results due to enhanced root systems. On the other hand, the seed of wheat under rainfed saline soil might not be fully filled with carbohydrates in extremely dry seasons, resulting in reduced grain yield, due to soil being too dry, and the soil salinity increases to unacceptable levels [26]. The superior performance for all genotypes in the 2021–2022 season compared to the 2020–2021 season in our study, therefore, could be attributed to higher rainfall during the entire growth period.

The response of wheat plant growth performance to soil nutrient availability is a dynamic process, which may vary between seasons because of the different weather conditions. Variations in weather conditions and soil nutrient availability during the entire growth period can impact plant development, altering the association among agronomic traits. In the current study, the grain yield correlated positively with straw weight, plant height, fertile spikelet per head, and grain number per head, indicating that a satisfactory grain yield depended on high aboveground biomass and spikelet fertility under coastal rainfed soil conditions. Indeed, the higher grain yield in 2021–2022 was associated with higher straw weight, plant height, fertile spikelet per head, and grain number per head relative to that in 2020–2021. On the other hand, the agronomic response to salt stress varies largely among different genotypes. Identifying suitable genotypes with high grain yield potential in coastal soils is, therefore, an essential prerequisite for large-scale cultivation. Genotype “Jimai 775”, which exhibited both a higher grain yield and a higher NAE than the others under the current field trial, is of promise to achieve satisfactory production benefits in coastal soil conditions. Due to the significant differences in the properties of saline soils in different coastal locations, it is necessary to explore the relationship between soil nutrient supply, soil properties, and nutrient utilization in order to develop a matched cultivation model for efficient nutrient utilization in wheat. The large-scale screening of diverse wheat genotypes for better agronomic performance and nutrient utilization efficiency through trials (multi-site and multi-season) under coastal saline soils is, hence, required in the next step, with the aim to identify suitable genotypes for sustainable production tailored to specific saline soil conditions.

### 3.2. Correlations Between Agronomic Traits and Nutrient Uptake Were Modified by N Supply

Differences in intrinsic genetic factors lead to variations in agronomic traits and nutrient accumulation among different genotypes. However, environmental factors and agronomic management practices such as fertilizer application can adaptively modify morphological development and nutrient uptake. Nutrient composition in plants can be altered by external environmental factors, such as salinity; a higher soil Na content led to a higher Na/K ratio, together with a differential elemental composition in wheat plants [27]. Similarly, correlations among grain Na, K, Ca, Mg, Zn, Fe, Se concentration varied between N supply treatments in the current study.

Genotype × environment interactions account for substantial variations in zinc utilization [28]. Consistently, the wheat grain Zn concentration was significantly different among genotypes in the current study. On the other hand, soil is the main Zn source for wheat grown without a fertilizer containing Zn, and soil properties, such as low soil moisture and organic matter and high pH and CaCO_3_, can hinder Zn translocation to grain [22]. Soil properties such as salt content and pH vary from site to site due to differences in irrigation conditions, groundwater depth, and agronomic management measures among different sites. Differential N supply results in contrasting soil environments among treatments, leading to variations in nutrient uptake and translocation in wheat plants. Grain Zn concentrations increased with higher N supply (N100 and N200 treatments) in the current study, consistent with the findings of Kutman et al. (2011) [17].

Genotypes with higher Fe or Zn concentrations could be planted extensively in coastal soil conditions for biofortification. The average grain Fe concentration of genotype LJJ803 (61.9 mg/kg) reached the Fe biofortification target. However, all eight genotypes had grain Zn concentrations below the biofortification target, with Jimai 106 showing the highest average value (33.1 mg/kg). Plant Zn uptake largely depends on soil properties such as available Zn content [29]. The experimental site in this study had a soil DTPA-Zn concentration of 0.9 mg/kg. Therefore, soil and/or foliar Zn applications are essential to achieve higher grain Zn concentrations in wheat grown in soils with low Zn availability.

Calcium is crucial for establishing plant cell walls, and the external application of Ca supplements on crops can alleviate abiotic stresses [30]. Magnesium is essential for normal plant growth as it is involved in chlorophyll biosynthesis. Increased P nutrition enhances plant Mg and Ca uptake [31,32], suggesting coordination among P, Mg, and Ca. In the current study, straw Mg, Ca, and P concentrations were consistently and positively correlated.

Although increased P input may decrease Zn uptake in wheat [33], wheat grain P and Zn concentrations tend to be positively correlated across diverse genotypes [34]. Moreover, elemental interactions play an important role in mediating the nutrient absorption of wheat, with Zn concentrations positively correlated with N, K, Mg, and Mn concentrations in wheat seedlings [20]. Grain Zn had a positive correlation with Ca, Cu, K, Mg (Shaukat, et al. 2021) [35]. Similarly, the uptake of Mg, P, K, and Ca positively influenced grain Zn accumulation, as the grain Zn concentration positively correlated with grain Mg, P, K, and Ca concentrations in the current study. Soil-available phosphorus was considered one of main soil factors affecting the grain iron content, and the grain iron content positively correlated with soil-available phosphorus in the study of Luo et al. (2025) [36]. Consistently, straw Fe concentrations positively correlated with straw P concentration in the current study, suggesting increased P uptake could facilitate Fe uptake from soil to plants.

Understanding the associations between agronomic traits and nutrient accumulation is crucial for wheat production under rainfed coastal soil conditions. Enhancements in agronomic performance may cause a negative impact on nutrient accumulation. For instance, increased grain yield was associated with higher grain and ear numbers but decreased concentrations of grain minerals, such as Fe, Mg, Na, P, and Zn [37]. In the current study, the grain Zn concentration positively correlated with the grain P concentration, grain Ca concentration, grain Mg concentration, straw weight, and 1000-kernel weight in the generalized regression equation model, suggesting the grain Zn concentration depended on both the plant developmental process and nutrient accumulation process within plants. A higher N supply level (N100, N200) increased the 1000-kernel weight of wheat grain relative to the control treatment, consistent with Klikocka et al. (2016) [38]. It can be deduced that the grain Zn concentration relied on post-anthesis nutrient remobilization from canopy to growing seeds; specifically, a higher grain Zn concentration caused by a higher N supply level (N100, N200) is associated with enhanced nutrient remobilization, including Ca, Mg, K, and P, together with improved 1000-kernel weight, in the current study. Interestingly, the grain Fe concentration positively correlated with straw P concentration and straw K concentration, while it negatively correlated with plant height and grain number per head in the regression model (Supplementary Table S2). This finding was in agreement with Hui et al. (2022) [33] and Luo et al. (2025) [36], wherein the grain Fe concentration was negatively correlated with yield and biomass. The decreased grain Fe concentration with increased biomass might be due to dilution effects. Selenium could play a role in the mitigation of plant salt stress [39,40], so it was not surprising that the regression model revealed a positive correlation between the grain Se concentration and grain Na concentrations in the current study. This might be due to more Se being required in some genotypes to deal with salt stress, resulting in a higher concentration of Se together with Na.

Soil salinity could affect the microbial composition in the rhizosphere and the nutrient translocation from soil to plants. Additionally, different N forms may have different effects on the rhizosphere process in relation to nutrient utilization in plants [41]. Figuring out the association among microbial change, nutrient utilization, and agronomic performance in wheat mediated by different N forms under coastal saline soils, therefore, deserves further exploration.

## 4. Materials and Methods

### 4.1. Experimental Setup

Field trials were conducted in a typical coastal field (37.57° N, 117.50° E) in Liupu town, Wudi, Shandong Province, China, over two consecutive seasons (2020–2021 and 2021–2022). This experimental site had a shallow groundwater table (0.8–1.2 m); the wheat growth was assumed to be influenced not only by salinity in topsoil layer but also by saline groundwater as roots penetrated into deeper soil layer. The topsoil (0–20 cm) at the experimental site had a pH of 8.1, ECe of 2.3 dS/m (as measured by extracts of saturated soil), 0.9 g N/kg, 6.8 mg Olsen-P/kg, 0.1 g exchangeable K/kg, and 0.9 mg DTPA-Zn/kg, 28.3 g total Ca/kg, 8.7 g total Mg/kg, 0.8 g Na/kg, 22.6 g total Fe/kg and 2.2 mg total Se/kg. The area experiences an average annual rainfall of 500–700 mm and annual evaporation of 1800–2000 mm. The wheat growth season usually starts in October and ends next June. The precipitation, average high temperature, average low temperature during 2020–2021 and 2021–2022 growing seasons are shown in Supplementary Figure S1. The experimental site received less precipitation in 2020–2021 growing season (118.2 mm) than that of 2021–2022 growing season (288.3 mm).

The field trial comprised eight newly bred winter wheat genotypes (Hongdi 95, Shannong 25, LJJ803, Jimai 775, LS018R, LS3666, Jimai 60, and Jimai 106) in Shandong province, China, and three N input levels with urea as the N source: N200 (200 kg/ha N, topdressed), N100 (100 kg/ha N, topdressed), and N0 (no N added). Each treatment had three replications, resulting in 72 plots (8 genotypes × 3 N input levels × 3 replications). Each plot, measuring 6 m^2^, received the specific N input and equal doses of P (150 kg/ha) using Ca(H_2_PO_4_)_2_ and K (120 kg/ha) using K_2_SO_4_. The fertilizers were applied for each plot before sowing. Seeds were sown manually in mid-October during both seasons, with a sowing density of 2.50 × 10^6^ seeds/ha for all genotypes. No irrigation was applied throughout the experiment. Plants were harvested in mid-June in both seasons.

### 4.2. Agronomic Performance

Agronomic traits for each genotype under different N supply levels were assessed. At maturity, 30 randomly selected plants from each plot were used to determine fertile spikelet number per head, non-fertile spikelet number per head, grain number per head, and 1000-kernel weight. Grain yield and straw weight were determined by harvesting and measuring all the plants within each plot.

### 4.3. Elemental Measurements

Plant samples were harvested, separated into straw and grain, oven-dried at 70 °C for 48 h, and ground into fine powder. A 50 mg subsample was digested with 13 mL HNO_3_ and 2 mL H_2_O_2_ in a tube, placed in a microwave workstation at 190 °C, with 15 min ramp to temperature (CEM MARS 6™ Microwave Digestion System, Matthews, NC, USA). After cooling (15 min), the digested samples were transferred to volumetric flask and diluted to 25 g, with deionized ultra pure water. The digest solution was adjusted to 50 mL before elemental concentrations (Na, K, Ca, Mg, P, Zn, Fe, Se) were determined using inductively coupled plasma optical emission spectrometry (iCAP PRO ICP-OES, Thermo Scientific, Waltham, MA, USA).

### 4.4. Nutrient Accumulation Parameters

Grain and straw Na, K, Ca, Mg, P, Zn, Fe, and Se concentrations were calculated as follows:

(C × V)/SW,

where C is the concentration of an individual element in the digest solution, V is the volume of the digest solution, and SW is the sample weight (50 mg).

Total Na, K, Ca, Mg, P, Zn, Fe, and Se accumulation in grain and straw was calculated as follows:

Total accumulation of an individual element in grain (or straw) = grain (or straw) weight × C_grain_ (or C_straw_),

where C_grain_ and C_straw_ are the concentrations (mg/kg) of individual elements in grain and straw, respectively.

### 4.5. Nitrogen Agronomic Efficiency

Nitrogen agronomic efficiency (NAE), defined as the amount of extra grain harvested per kilogram of N applied to a grain crop relative to control (without N application), was calculated as follows:

NAE (kg/kg) = (Grain yield in treatment − Grain yield in control)/(N input amount)

### 4.6. Statistical Analysis

A Season × N supply × Genotype interaction model was analyzed by ANOVA using SPSS software 16.0 (IBM, Armonk, NY, USA). All investigated parameters were tested for normality and homogeneity by Kolmogorov–Smirnov tests in SPSS software before subjecting to ANOVA. A mixed linear model accounted for fixed effects (season, genotype, and N supply) and random effects (block and measured replication). Multiple comparisons (Tukey) of measured parameters were conducted among different N supply levels for the same genotype within the same season, as well as among genotypes, with a significance level (α) set at 0.05. Stepwise multiple regression equations were constructed using investigated traits, including agronomic traits and nutrient concentrations, in SPSS software. For the regression equation model, grain Zn (or Fe, Se) concentration was used as the dependent variable, while other traits were used as independent variables. Principal component analysis (PCA) was performed using Origin 2018 software (Origin Lab, Northampton, MA, USA), while Pearson’s correlation analysis was conducted using SPSS software to examine relationships among variables.

## 5. Conclusions

In summary, this study investigated the impact of the N supply on the agronomic performance and nutrient accumulation in eight wheat genotypes grown under rainfed coastal soil conditions. Season, genotype, and N supply significantly influenced agronomic traits and nutrient accumulation parameters, including grain and straw Na, K, Ca, Mg, P, Zn, Fe, and Se concentrations. Increasing the N supply enhanced agronomic performance and grain Zn and Fe concentrations but reduced the grain Se concentration. N supply had a significant impact on the correlation among grain elemental accumulation. A better fertilizer management approach targeting wheat genotypes with higher Zn, Fe, and Se uptake holds promise for achieving the biofortification target in wheat grown under coastal soil conditions. This study underscores the importance of nutrient management strategies tailored to specific genotypes and environmental conditions to enhance crop productivity and nutritional quality. N supply could affect nutrient metabolism and plant development in wheat, hence changing the association among elemental accumulation and agronomic performance. Both the N supply level and genotypic differences should be taken into consideration for improved nutrient utilization in wheat cultivated in coastal saline soils and other similar challenging agricultural environments.

## Figures and Tables

**Table 1:** Plant height, fertile spikelet number per head, grain number per head, 1000-grain weight, grain yield, and straw weight under three N supply levels (N0, N100, N200) in the 2020–2021 and 2021–2022 seasons.

Season	N Supply	PH (cm)	FSNH	GNH	TKW (g)	GY (kg/ha)	SW (kg/ha)
2020–2021	N0	53.0 ± 4.4 ^c^	15.4 ± 0.7 ^a^	41.6 ± 2.7 ^b^	40.1 ± 2.2 ^c^	4013.7 ± 611.8 ^c^	4404.5 ± 602.1 ^c^
	N100	54.3 ± 4.3 ^b^	15.6 ± 0.6 ^a^	41.4 ± 3.4 ^b^	40.5 ± 2.2 ^b^	4706.6 ± 710.2 ^b^	5136.8 ± 775.8 ^b^
	N200	57.1 ± 5.2 ^a^	15.8 ± 0.9 ^a^	44.6 ± 2.0 ^a^	40.8 ± 2.3 ^a^	5782.3 ± 890.0 ^a^	6110.8 ± 1009.9 ^a^
2021–2022	N0	55.0 ± 4.4 ^b^	16.0 ± 0.8 ^b^	42.6 ± 3.3 ^b^	41.2 ± 2.3 ^c^	4158.2 ± 619.7 ^c^	4120.3 ± 658.1 ^c^
	N100	57.1 ± 5.7 ^a^	16.2 ± 0.7 ^a^	43.3 ± 3.8 ^b^	41.5 ± 2.4 ^b^	4921.2 ± 784.8 ^b^	5095.3 ± 1068.2 ^b^
	N200	58.9 ± 4.6 ^a^	16.4 ± 1.0 ^a^	46.6 ± 2.4 ^a^	41.9 ± 2.4 ^a^	6073.9 ± 916.7 ^a^	6096.9 ± 1326.6 ^a^

Note: Different letters indicate statistically significant difference (*p* < 0.05). Abbreviation: FSNH, fertile spikelet number per head; GNH, grain number per head; GY, grain yield; PH, plant height; SW, straw weight; TKW, 1000-kernel weight.

**Table 2:** Multiple comparisons of agronomic traits of eight wheat genotypes at maturity.

Growing Season	Genotype	PH (cm)	FSNH	GNH	TKW (g)	GY (kg/ha)	SW (kg/ha)
2020–2021	Hongdi95	49.6 ± 0.9 ^d^	15.1 ± 0.5 ^cd^	41.0 ± 3.3 ^bc^	40.3 ± 0.6 ^bc^	3667.8 ± 558.4 ^d^	3964.1 ± 723.4 ^d^
	Jimai106	50.7 ± 1.3 ^d^	15.9 ± 0.7 ^b^	44.2 ± 1.2 ^a^	43.3 ± 0.9 ^a^	4759.1 ± 1176.8 ^c^	5219.7 ± 1245.3 ^bc^
	Jimai60	62.9 ± 2.2 ^a^	15.5 ± 0.7 ^bc^	44.2 ± 2.1 ^a^	43.5 ± 0.5 ^a^	5457.7 ± 781.3 ^ab^	5730.2 ± 650.4 ^ab^
	Jimai775	54.6 ± 2.7 ^c^	16.4 ± 0.7 ^a^	44.4 ± 3.9 ^a^	36.7 ± 0.8 ^e^	5782.7 ± 1363.7 ^a^	6135.1 ± 1472.2 ^a^
	LJJ803	55.4 ± 5.0 ^bc^	15.5 ± 0.6 ^bc^	38.6 ± 4.4 ^c^	38.8 ± 0.9 ^d^	4572.5 ± 906.3 ^c^	5166.9 1118.3 ^bc^
	LS018R	54.4 ± 3.9 ^c^	14.8 ± 0.4 ^d^	40.4 ± 3.4 ^c^	40.6 ± 0.6 ^b^	4836.0 ± 372.7 ^c^	5148.5 ± 357.9 ^bc^
	LS3666	56.5 ± 3.9 ^b^	15.8 ± 0.7 ^b^	43.2 ± 1.0 ^ab^	39.7 ± 1.3 ^c^	4591.5 ± 549.2 ^c^	4904.5 ± 514.7 ^c^
	Shannong25	54.3 ± 3.6 ^c^	15.7 ± 0.5 ^b^	42.9 ± 3.2 ^ab^	40.8 ± 0.8 ^b^	5006.3 ± 988.3 ^bc^	5469.8 ± 769.9 ^abc^
2021–2022	Hongdi95	51.6 ± 1.7 ^d^	15.6 ± 0.6 ^c^	43.1 ± 4.0 ^bcd^	41.2 ± 1.0 ^bc^	3865.3 ± 585.8 ^c^	4033.9 ± 860.0 ^b^
	Jimai106	53.1 ± 1.7 ^d^	16.4 ± 0.7 ^b^	46.0 ± 1.3 ^ab^	44.9 ± 1.0 ^a^	4817.0 ± 1229.7 ^b^	5128.9 ± 1028.4 ^ab^
	Jimai60	64.9 ± 3.4 ^a^	16.1 ± 0.6 ^bc^	46.4 ± 2.2 ^a^	44.2 ± 0.5 ^a^	5696.0 ± 807.7 ^a^	5820.1 ± 979.5 ^a^
	Jimai775	57.7 ± 3.6 ^bc^	17.3 ± 0.7 ^a^	45.8 ± 4.4 ^ab^	37.3 ± 0.9 ^e^	6081.7 ± 1444.8 ^a^	5078.9 ± 1484.1 ^ab^
	LJJ803	57.6 ± 5.8 ^bc^	16.0 ± 0.8 ^bc^	41.2 ± 3.3 ^d^	39.8 ± 1.2 ^d^	4770.6 ± 949.7 ^b^	5655.9 ± 1817.6 ^a^
	LS018R	56.0 ± 3.7 ^c^	15.6 ± 0.5 ^c^	41.7 ± 3.7 ^cd^	42.0 ± 0.7 ^b^	5120.7 ± 413.2 ^b^	5135.2 ± 1060.2 ^ab^
	LS3666	58.4 ± 4.2 ^b^	16.4 ± 0.8 ^b^	44.6 ± 1.8 ^abc^	40.9 ± 1.3 ^c^	4867.4 ± 527.3 ^b^	4297.9 ± 707.8 ^b^
	Shannong25	56.4 ± 3.5 ^bc^	16.2 ± 0.4 ^bc^	44.5 ± 3.8 ^abc^	41.7 ± 1.1 ^bc^	5190.3 ± 1210.6 ^b^	5682.6 ± 1496.8 ^a^

Note: Different letters within the same column indicate significant differences at the 0.05 level. Abbreviation: FSNH, fertile spikelet number per head; GNH, grain number per head; GY, grain yield; PH, plant height; SW, straw weight; TKW, 1000-kernel weight.

**Table 3:** Multiple comparisons of grain elemental concentration of eight wheat genotypes at maturity.

Growing Season	Genotype	Na (mg/kg)	K (g/kg)	Ca (mg/kg)	Mg (g/kg)	P (g/kg)	Zn (mg/kg)	Fe (mg/kg)	Se (µg/kg)
2020–2021	Hongdi95	51.7 ± 18.5 ^b^	4.6 ± 0.5 ^a^	524.3 ± 41.2 ^a^	1.6 ± 0.1 ^ab^	4.4 ± 0.5 ^a^	28.4 ± 2.8 ^ab^	51.9 ± 3.1 ^bc^	55.8 ± 6.7 ^ab^
	Jimai106	43.3 ± 3.5 ^c^	4.2 ± 0.4 ^abc^	501.8 ± 52.8 ^ab^	1.5 ± 0.1 ^bc^	3.9 ± 0.4 ^b^	30.2 ± 4.6 ^a^	55.1 ± 4.5 ^b^	50.6 ± 7.9 ^c^
	Jimai60	64.3 ± 14.1 ^a^	4.4 ± 0.3 ^a^	441.8 ± 29.8 ^d^	1.4 ± 0.1 ^c^	3.4 ± 0.5 ^c^	23.1 ± 2.9 ^de^	50.1 ± 3.8 ^c^	54.2 ± 7.1 ^bc^
	Jimai775	45.2 ± 5.2 ^c^	4.0 ± 0.4 ^bc^	487.9 ± 59.8 ^abc^	1.6 ± 0.1 ^ab^	3.4 ± 0.5 ^c^	25.1 ± 4.5 ^cd^	48.4 ± 4.8 ^cd^	57.3 ± 5.4 ^ab^
	LJJ803	49.9 ± 14.1 ^b^	4.4 ± 0.4 ^a^	511.9 ± 40.3 ^ab^	1.6 ± 0.2 ^a^	4.0 ± 0.5 ^b^	26.5 ± 4.6 ^bc^	63.6 ± 15.3 ^a^	57.2 ± 3.9 ^ab^
	LS018R	35.3 ± 4.4 ^d^	4.3 ± 0.4 ^ab^	486.4 ± 43.1 ^abcd^	1.5 ± 0.2 ^bc^	3.3 ± 0.5 ^c^	23.8 ± 2.6 ^d^	49.8 ± 2.1 ^c^	56.8 ± 3.9 ^ab^
	LS3666	52.2 ± 13.6 ^b^	4.2 ± 0.3 ^abc^	449.5 ± 82.6 ^cd^	1.4 ± 0.1 ^c^	3.2 ± 0.2 ^cd^	21.3 ± 1.2 ^e^	44.9 ± 2.4 ^d^	58.6 ± 9.1 ^ab^
	Shannong25	44.8 ± 9.4 ^c^	3.9 ± 0.3 ^c^	478.4 ± 36.6 ^bcd^	1.4 ± 0.1 ^c^	2.9 ± 0.2 ^d^	23.5 ± 3.8 ^de^	49.6 ± 2.7 ^c^	60.0 ± 10.4 ^a^
2021–2022	Hongdi95	47.6 ± 17.2 ^b^	4.1 ± 0.4 ^a^	489.7 ± 31.9 ^a^	1.4 ± 0.1 ^ab^	4.1 ± 0.4 ^a^	35.1 ± 3.5 ^ab^	48.5 ± 3.2 ^bc^	51.8 ± 6.2 ^bc^
	Jimai106	39.7 ± 3.6 ^c^	3.9 ± 0.4 ^b^	470.1 ± 54.8 ^a^	1.3 ± 0.1 ^bc^	3.6 ± 0.2 ^b^	35.9 ± 3.4 ^a^	51.9 ± 3.0 ^b^	47.0 ± 5.8 ^d^
	Jimai60	58.5 ± 11.1 ^a^	3.9 ± 0.2 ^ab^	406.8 ± 16.8 ^c^	1.3 ± 0.1 ^c^	3.2 ± 0.5 ^c^	30.4 ± 6.2 ^cd^	46.4 ± 2.9 ^cd^	49.5 ± 5.5 ^cd^
	Jimai775	41.5 ± 4.8 ^c^	3.6 ± 0.4 ^c^	453.9 ± 57.2 ^ab^	1.4 ± 0.1 ^ab^	3.2 ± 0.5 ^c^	31.2 ± 6.9 ^bc^	45.2 ± 4.2 ^cd^	53.1 ± 4.6 ^abc^
	LJJ803	48.1 ± 13.6 ^b^	4.1 ± 0.3 ^ab^	481.6 ± 44.5 ^a^	1.5 ± 0.2 ^a^	3.7 ± 0.5 ^b^	32.8 ± 5.3 ^abc^	60.4 ± 1.3 ^a^	53.8 ± 2.5 ^abc^
	LS018R	33.2 ± 3.7 ^d^	3.9 ± 0.4 ^ab^	454.8 ± 47.3 ^ab^	1.3 ± 0.2 ^bc^	3.0 ± 0.4 ^c^	30.8 ± 5.6 ^bc^	47.1 ± 3.1 ^cd^	52.6 ± 3.3 ^bc^
	LS3666	48.9 ± 13.0 ^b^	3.9 ± 0.2 ^c^	427.9 ± 79.2 ^bc^	1.3 ± 0.1 ^c^	3.0 ± 0.2 ^cd^	26.1 ± 2.5 ^d^	42.9 ± 2.6 ^d^	55.3 ± 7.8 ^ab^
	Shannong25	41.5 ± 8.5 ^c^	3.5 ± 0.3 ^c^	453.6 ± 38.5 ^ab^	1.3 ± 0.2 ^bc^	2.8 ± 0.3 ^d^	30.4 ± 5.2 ^cd^	47.5 ± 3.6 ^c^	57.2 ± 8.9 ^a^

Note: Different letters within the same column indicate significant differences at the 0.05 level.

**Table 4:** Nitrogen agronomic efficiency of eight wheat genotypes grown under N100 and N200 supply levels.

Growing Season	Genotype	NAE (kg/kg)	
		N100	N200
2020–2021	Hongdi95	4.6 ± 0.3 ^c^	6.0 ± 0.4 ^b^
	Jimai106	6.1 ± 1.9 ^c^	13.0 ± 0.8 ^ab^
	Jimai60	1.5 ± 0.2 ^d^	7.6 ± 0.5 ^b^
	Jimai775	18.7 ± 2.1 ^a^	15.4 ± 0.9 ^a^
	LJJ803	8.0 ± 0.7 ^c^	10.0 ± 0.6 ^b^
	LS018R	2.1 ± 0.6 ^d^	3.3 ± 0.7 ^c^
	LS3666	0.5 ± 0.1 ^d^	5.1 ± 0.3 ^c^
	Shannong25	14.9 ± 1.5 ^b^	10.2 ± 2.0 ^b^
2021–2022	Hongdi95	6.3 ± 0.9 ^c^	4.2 ± 1.7 ^cd^
	Jimai106	13.4 ± 1.7 ^ab^	6.4 ± 0.9 ^c^
	Jimai60	7.8 ± 1.7 ^bc^	3.5 ± 0.4 ^cd^
	Jimai775	16.0 ± 0.6 ^a^	20.4 ± 4.7 ^a^
	LJJ803	10.4 ± 1.8 ^b^	8.2 ± 2.4 ^c^
	LS018R	3.8 ± 1.6 ^c^	3.1 ± 1.1 ^cd^
	LS3666	5.4 ± 0.3 ^c^	2.2 ± 0.6 ^d^
	Shannong25	13.6 ± 0.7 ^ab^	14.2 ± 1.4 ^b^

Note: Different letters within the same column indicate significant differences at the 0.05 level.

## Data Availability

Data are contained within the article and supplementary material. Further inquiries can be directed to the corresponding author.

## Supplementary Materials

The following supporting information can be downloaded at: https://www.mdpi.com/article/10.3390/plants14071022/s1, Table S1. Three-way analysis of variance of the effects of season, N supply, and genotype on investigated agronomic traits. Table S2. Regression model between grain Zn, Fe, Se concentrations and agronomic traits and other elemental concentrations. Figure S1. Precipitation and temperature during 2020–2021 (A) and 2021–2022 (B) growth seasons.
