# Supplementary material for: Agronomic Traits, Nutrient Accumulation, and Their Correlations in Wheat, as Affected by Nitrogen Supply in Rainfed Coastal Saline Soils
Source: Plants (Basel). 2025 Mar 25;14(7):1022. doi: 10.3390/plants14071022 (PMC11990352; doi:10.3390/plants14071022)
Supplement: Supplementary file 1 [file plants-14-01022-s001.zip › plants-3519931-supplementary.pdf]

Agronomic Traits, Nutrient Accumulation, and Their Correlations in Wheat, as Affected by Nitrogen Supply in Rainfed Coastal Saline Soils

Yan Li [1], Shuaipeng Zhao [1,2,3], Guolan Liu [1,2,3], Jian Li [1,2,3], Kadambot H. M. Siddique [4] and Deyong Zhao [1,2,3,*]

[1] College of Biological and Pharmaceutical Engineering, Shandong University of Aeronautics, Binzhou 256603, China; 23021201005@sdua.edu.cn (Y.L.)
[2] Shandong Key Laboratory of Eco-Environmental Science for Yellow River Delta, Shandong University of Aeronautics, Binzhou 256603, China
[3] Shandong Engineering and Technology Research Center for Fragile Ecological Belt of Yellow River Delta, Shandong University of Aeronautics, Binzhou 256603, China
[4] The UWA Institute of Agriculture, The University of Western Australia, Perth, WA 6001, Australia; kadambot.siddique@uwa.edu.au
* Correspondence: dyzhao@sdua.edu.cn

Supplementary Table S1. Three-way analysis of variance of the effects of season, N supply, and genotype on investigated agronomic traits

Source of variation	PH (cm)	FSNH	GNH	TKW (g)	GY (kg/ha)	SW (kg/ha)
Season	<0.01	<0.01	<0.01	<0.01	<0.01	0.35
N supply	<0.01	<0.01	<0.01	<0.01	<0.01	<0.01
Genotype	<0.01	<0.01	<0.01	<0.01	<0.01	<0.01
Season × N supply	0.15	0.90	0.63	0.74	0.53	0.07
Season × Genotype	0.85	0.46	0.99	0.37	0.98	0.03
N supply × Genotype	<0.01	<0.01	<0.01	<0.01	<0.01	0.02
Season × N supply × Genotype	<0.01	0.99	0.99	0.79	0.99	0.44

Note: The value shown was the probability of F value. Abbreviations: PH, plant height; GY, grain yield; SW, straw weight; GNH, grain number per head; FSNH, fertile spikelet number per head; TKW, 1000-kernel weight.

Supplementary Table S2. Regression model between grain Zn, Fe, Se concentrations and agronomic traits and other elemental concentrations

Growing season	N treatment	Equation
2020–2021	N0	(1) $Y_{Zn\_Grain} = 0.004X_{P\_Grain} + 0.010X_{Mg\_Grain} + 0.118X_{Se\_Straw} - 16.230$
		(2) $Y_{Fe\_Grain} = -3.558X_{GNH} - 7.195X_{Zn\_Straw} - 0.034X_{Mg\_Grain} - 2.264X_{TKW} - 0.004X_{SW} + 402.986$
		(3) $Y_{Se\_Grain} = -0.097X_{Fe\_Straw} + 0.302X_{PH} + 0.010X_{K\_Grain} + 0.001X_{K\_Straw} + 0.021X_{Ca\_Grain} - 12.485$
	N100	(1) $Y_{Zn\_Grain} = 0.003X_{P\_Grain} + 0.458X_{Fe\_Grain} - 8.818$
		(2) $Y_{Fe\_Grain} = 0.535X_{Zn\_Grain} - 0.381X_{PH} + 56.774$
		(3) $Y_{Se\_Grain} = 0.636X_{Na\_Grain} - 0.008X_{K\_Grain} + 0.033X_{P\_Straw} + 44.317$
	N200	(1) $Y_{Zn\_Grain} = 0.782X_{Fe\_Grain} - 0.113X_{Na\_Grain} + 0.794X_{Zn\_Straw} - 13.421$
		(2) $Y_{Fe\_Grain} = 0.885X_{Zn\_Grain} + 0.074X_{Na\_Grain} + 0.003X_{P\_Grain} + 14.528$
		(3) $Y_{Se\_Grain} = -0.727X_{Se\_Straw} + 0.025X_{Mg\_Straw} + 0.729X_{Zn\_Straw} + 99.073$
	Combined N0, N100, and N200	(1) $Y_{Zn\_Grain} = 0.005X_{P\_Grain} + 0.025X_{Ca\_Grain} + 0.001X_{GY} - 10.214$
		(2) $Y_{Fe\_Grain} = -0.920X_{GNH} - 0.014X_{Mg\_Straw} + 0.021X_{P\_Straw} + 101.986$
		(3) $Y_{Se\_Grain} = -0.244X_{Se\_Straw} - 0.542X_{PH} + 0.156X_{Na\_Grain} + 105.618$
2021–2022	N0	(1) $Y_{Zn\_Grain} = 0.008X_{P\_Grain} - 0.006X_{Mg\_Straw} + 11.632$
		(2) $Y_{Fe\_Grain} = -2.790X_{GNH} - 4.416X_{Zn\_Straw} + 0.332X_{Na\_Grain} + 184.671$
		(3) $Y_{Se\_Grain} = -0.089X_{Fe\_Straw} + 72.295$
	N100	(1) $Y_{Zn\_Grain} = 0.006X_{P\_Grain} + 12.670$
		(2) $Y_{Fe\_Grain} = 0.063X_{Ca\_Grain} + 16.797$
		(3) $Y_{Se\_Grain} = 0.114X_{P\_Straw} - 3.074X_{Zn\_Straw} + 33.843$
	N200	(1) $Y_{Zn\_Grain} = 1.020X_{Fe\_Grain} + 0.007X_{Ca\_Grain} - 34.0624$
		(2) $Y_{Fe\_Grain} = 0.724X_{Zn\_Grain} - 0.009X_{Ca\_Straw} + 0.027X_{Fe\_Straw} + 42.911$
		(3) $Y_{Se\_Grain} = -0.127X_{Fe\_Straw} + 1.546X_{Zn\_Straw} + 64.592$
	Combined N0, N100, and N200	(1) $Y_{Zn\_Grain} = 0.007X_{P\_Grain} + 0.002X_{GY} - 0.262X_{PH} - 1.567X_{FSNH} + 37.591$
		(2) $Y_{Fe\_Grain} = -0.954X_{GNH} - 0.008X_{Mg\_Straw} + 0.279X_{Zn\_Grain} + 91.642$
		(3) $Y_{Se\_Grain} = -0.825X_{TKW} + 86.838$
Combined N0, N100, and N200 treatments of both 2020–2021 and 2021–2022 seasons	/	(1) $Y_{Zn\_Grain} = 0.003X_{P\_Grain} + 0.013X_{Ca\_Grain} + 0.001X_{SW} - 0.377X_{PH} + 0.374X_{Zn\_Straw} + 0.5X_{TKW} + 0.008X_{Mg\_Grain} - 13.578$
		(2) $Y_{Fe\_Grain} = 0.033X_{P\_Straw} + 0.006X_{K\_Grain} + 2.656X_{FSN} - 0.867X_{GN} - 0.014X_{Mg\_Straw} - 0.494X_{PH} + 56.263.$
		(3) $Y_{Se\_Grain} = -0.330X_{Se\_Straw} - 0.994X_{PH} + 0.119X_{Na\_Grain} - 0.012X_{P\_Grain} + 0.032X_{Mg\_Grain} + 0.006X_{K\_Grain} - 0.045X_{Ca\_Grain} + 0.004X_{Na\_Straw} + 124.646.$

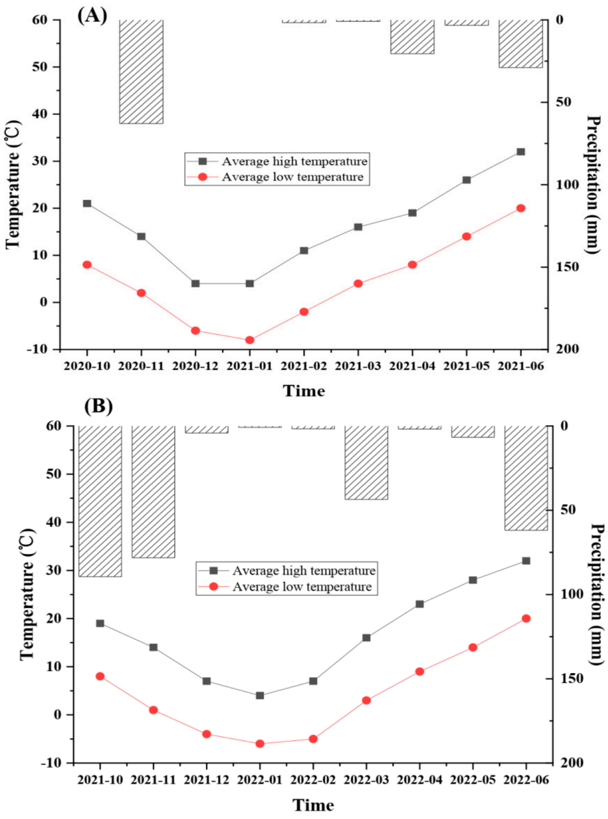

Supplementary Figure S1. Precipitation and temperature during 2020-2021 (A) and 2021-2022 (B) growth seasons
